# The role of secreted Hsp90α in HDM-induced asthmatic airway epithelial barrier dysfunction

**DOI:** 10.1186/s12890-019-0938-z

**Published:** 2019-11-20

**Authors:** Cuiping Ye, Chaowen Huang, Mengchen Zou, Yahui Hu, Lishan Luo, Yilan Wei, Xuan Wan, Haijin Zhao, Wei Li, Shaoxi Cai, Hangming Dong

**Affiliations:** 1 Chronic Airways Diseases Laboratory, Department of Respiratory and Critical Care Medicine, Nan Fang Hospital, Southern Medical University, Guangzhou, 510515 People’s Republic of China; 2 Department of Respiratory Medicine, Jiangmen Central Hospital, Jiangmen, Guangdong 529030 People’s Republic of China; 3 Department of Endocrinology and Metabolism, Nan Fang Hospital, Southern Medical University, Guangzhou, 510515 People’s Republic of China; 4 Department of Dermatology and the Norris Comprehensive Cancer Centre, University of Southern California Keck, Medical Centre, Los Angeles, CA 90033 USA

**Keywords:** Asthma, Epithelial barrier, Secreted Hsp90α, 1G6-D7, HDM

## Abstract

**Background:**

The dysfunction of airway epithelial barrier is closely related to the pathogenesis of asthma. Secreted Hsp90α participates in inflammation and Hsp90 inhibitor protects endothelial dysfunction. In the current study, we aimed to explore the role of secreted Hsp90α in asthmatic airway epithelial barrier function.

**Methods:**

Male BALB/c mice were sensitized and challenged with HDM to generate asthma model. The 16HBE and Hsp90α-knockdown cells were cultured and treated according to the experiment requirements. Transepithelial Electric Resistance (TEER) and permeability of epithelial layer in vitro, distribution and expression of junction proteins both in vivo and in vitro were used to evaluate the epithelial barrier function. Western Blot was used to evaluate the expression of junction proteins and phosphorylated AKT in cells and lung tissues while ELISA were used to evaluate the Hsp90α expression and cytokines release in the lung homogenate.

**Results:**

HDM resulted in a dysfunction of airway epithelial barrier both in vivo and in vitro, paralleled with the increased expression and release of Hsp90α. All of which were rescued in Hsp90α-knockdown cells or co-administration of 1G6-D7. Furthermore, either 1G6-D7 or PI3K inhibitor LY294002 suppressed the significant phosphorylation of AKT, which caused by secreted and recombinant Hsp90α, resulting in the restoration of epithelial barrier function.

**Conclusions:**

Secreted Hsp90α medicates HDM-induced asthmatic airway epithelial barrier dysfunction via PI3K/AKT pathway, indicating that anti-secreted Hsp90α therapy might be a potential treatment to asthma in future.

## Background

Asthma is known to be a chronic airway disease, which is characterized by inflammation, shedding of airway epithelial cells (AECs) and airway remodeling [1]. Chronic inflammation exacerbates intensively once the airway is exposed to antigens. As the first defensive barrier between lung and outer environment, AECs and their contacts play important roles in defense, antigen presentation and quick response to different stimulation [2].

The tight junctions (TJs) are on the surface of AECs, encircling the subapical regions of lateral cell membranes to regulate permeability and restrict lateral movement of the cell membrane. The functions of TJs rely on the interaction of protein complexes [3]. Below the TJs are the adherens junctions (AJs) composing of E-cadherin, β-catenin, p120 and plakoglobulin. Studies had shown that E-cadherin and β-catenin acted as not only important barrier proteins to anchor AECs, but also crucial signaling proteins for immune response [4–6].

Hsp90 is a member of HSPs family and is defined as molecular chaperones for a long time [7]. Hsp90 expresses constitutively in eukaryotes and its expression is upregulated in various situations such as in stress, inflammation and adverse stimulations. Hsp90α, one of the four subtypes of Hsp90, exists not only in cytoplasm but also on the surface of certain cells [8, 9]. Evidences showed that Hsp90α is actively secreted to intercellular and tissue space for promoting wound healing, inflammatory mediation, invasion and migration. Hsp90α and Hsp90β have different and non-exchangeable functions during wound healing [10]. Asthmatic AECs are always in a damage and self-repair period, yet the role of secreted Hsp90α in asthma is still unknown.

Our previous studies focused on the disruptions of TJs and AJs in asthma, in which we demonstrated that TDI and HDM could cause dysfunctions of TJs and AJs via VEGF pathway or AKT pathway [11–14]. We found HDM promoted the secretion of Hsp90α in preliminary experiments, and it has not been confirmed whether the secreted Hsp90α plays an important role in asthma. In this study, we evaluated the secretion of Hsp90α and the expression of epithelial barrier proteins. Our data show that secreted Hsp90α is upregulated at the protein levels in response to HDM in mice and 16HBE cells. We also found that secreted Hsp90α contributes to HDM-induced airway epithelial barrier dysfunction and 1G6-D7 prevents this HDM-induced disruption.

## Methods

### Animals and reagents

All the animal experiments were approved by the Committee on the Ethics of Animal Experiments of Southern Medical University in Guangzhou, China and performed under standard guidelines for the Care and Use of Laboratory Animals. SPF BALB/c mice (male, 6-week old, 20–24 g) were purchased from Southern Medical University. The mice were housed in the laboratory with a 12:12-h light/dark cycle at 24°C in an atmosphere of 40–70% humidity. Food and water were sterilized and all experiments involving animals complied with the ARRIVE guidelines. HDM was purchased from ALK-Abello A/S, methacholine was obtained from Sigma-Aldrich and 1G6-D7 (specific anti-secreted Hsp90α monoclonal antibody, mAb) was developed and contributed by Wei Li’s laboratory [15, 16].

### HDM-induced asthma and 1G6-D7 administration

#### The establishment and evaluation of HDM-induced asthmatic animal model

BALB/c mice were randomly distributed to 4 groups(*n* = 8 per group): (1) control group; (2) 1G6-D7 group; (3) HDM group; (4) 1G6-D7 + HDM group. In this study mice were delivered to intranasal sevoflurane-anesthesia (Maruishi Pharmaceutical Co. Ltd.), then respectively administered with 10 μl of PBS, HDM(400 U/mouse a day), 1G6-D7 (0.1 μg/μl, 10 μl, dissolved in PBS) or 1G6-D7 + HDM daily for 5 consecutive days, during 8 weeks period. All groups were administered via intranasal inhalation. Furthermore, in the 1G6-D7 + HDM group, mice were pretreated with 1G6-D7 30 min before administration of HDM and the concentrations were described above.

#### RNAi delivery system, cell culture and treatment

16HBE cells were purchased from Fuxiang Biological Technology Co. Ltd., ATCC, USA. We used the RNAi delivery system to knockdown the Hsp90α (Han Bio, Shanghai, China). The selected RNAi sequence against Hsp90α was 5′-GGAAAGAGCTGCATATTAA-3′ [15], RNAi was cloned into the lentiviral RNAi delivery vector and the gene transduction efficiency of infected 16HBE cells was monitored based on the co-expressed GFP gene marker in the same vector under a fluorescent microscope. When reached 85% confluence, normal 16HBE cells were maintained in serum-free medium for 12 h, then treated with HDM (400 U/ml) with or without 1G6-D7 (25 μg/ml). The Hsp90α-knockdown 16HBE cells were treated with HDM (400 U/ml) or human recombinant Hsp90α (hrHsp90α, 10 μg/ml, Stressmarq Biosciences Inc.). After 24 h, cells were harvested for cell lysates preparation for 12 h. Condition media was collected to investigate the amount of Hsp90α and Hsp90β. In a further experiment, normal 16HBE cells were treated with HDM (400 U/ml) or hrHsp90α (10 μg/ml), with or without LY294002 (10 μM, Cell signaling technology, CST).

### The measurement of epithelial barrier function and immunofluorescence

The measurements of transepithelial electrical resistance (TEER), Permeability (FITC-dextran) and immunofluorescence of E-cadherin and β-catenin were performed as described in our previous study [11]. Primary antibodies anti-E-cadherin, anti-β-catenin and FITC (green or red)-linked anti-rabbit IgG were obtained from Santa Cruz Biotechnology, USA. 4′, 6-diamidino-2-phenylindole dihydrochloride (DAPI) was obtained from Sigma-Aldrich.

### Airway resistance to methacholine, mice euthanasia and necropsy

24 h after the last administration, lung resistance (RL) was assessed to evaluate the airway resistance. Mice were placed in a barometric plethysmo-graphic chamber (Buxco Electronics, Troy, NY) and provocated with vehicle (PBS), followed by increasing concentrations of methacholine (6.25 mg/ml, 12.5 mg/ml, 25 mg/ml, and 50 mg/ml) via nebulizer (Buxco Electronics, Inc., Troy, NY) for 3 min. RL and other data were monitored at the same time. The detail protocols of mice sacrifice, anesthesia, sample collection and sample storage were executed as described in our previous study [13].

### Western blot analysis

The supernatants of cell and the completely homogenized right lung tissue samples were collected and boiled with standard SDS sample buffer. The secreted Hsp90α(Calbiochem, Merck.), secreted Hsp90β(Stressmarq Biosciences Inc.) in conditioned media, Hsp90α, Hsp90β, p-AKT (Thr 308, CST), pan-AKT (CST), p-ERK1/2(CST), ERK1/2(CST), p-JNK (CST), JNK (CST), E-cadherin (Santa Cruz.), β-catenin (Santa Cruz.), occluding (Santa Cruz.), claudin1-2(Santa Cruz.) in cell lysate and tissue lysate were analyzed.

### Elisa

As previously described, mice were sacrificed with pentobarbital (100 mg/kg, i.p.) 1 day after the last airway challenge. Blood samples were allowed to rest for 2 h at room temperature, then centrifuged (3000×g, 20 min), and supernatants were harvested and stored at − 80 °C. IgE (BD Bioscience.), Hsp90α(Cloud-Clone Corp.) in serum and Hsp90α, IL-4 (affymetrix, eBioscience.), IL-5 (affymetrix, eBioscience.), IL-13 (affymetrix, eBioscience.), IL-33 (affymetrix, eBioscience.), IFN-γ (affymetrix, eBioscience.) in BALF were measured by ELISA following the manufacturer’s instructions.

### Histopathology and immunohistochemistry

Left lungs were gently infused with 10% Paraformaldehyde to fully inflate all lobes (inflation was judged visually) and immersed in Paraformaldehyde for at least 24 h, then fixed, paraffin-embedded, cut in 4-μm sections, and stained with hematoxylin and eosin (H&E) for blinded histopathologic assessment. Immunohistochemistry for Hsp90α, E-cadherin and β-catenin were performed as previously study described [13].

### Statistical analysis

Statistical analysis was computed using SPSS (version 19.0). The variables were expressed as mean ± standard deviation. One-way ANOVA accompanied by Bonferonni post hoc test for multiple comparisons were utilized to compare differences among groups. *P* < 0.05 was considered as statistical significance.

## Results

### Secreted Hsp90α was released in asthmatic mice and 1G6-D7 alleviated AHR

**Fig. 1:**
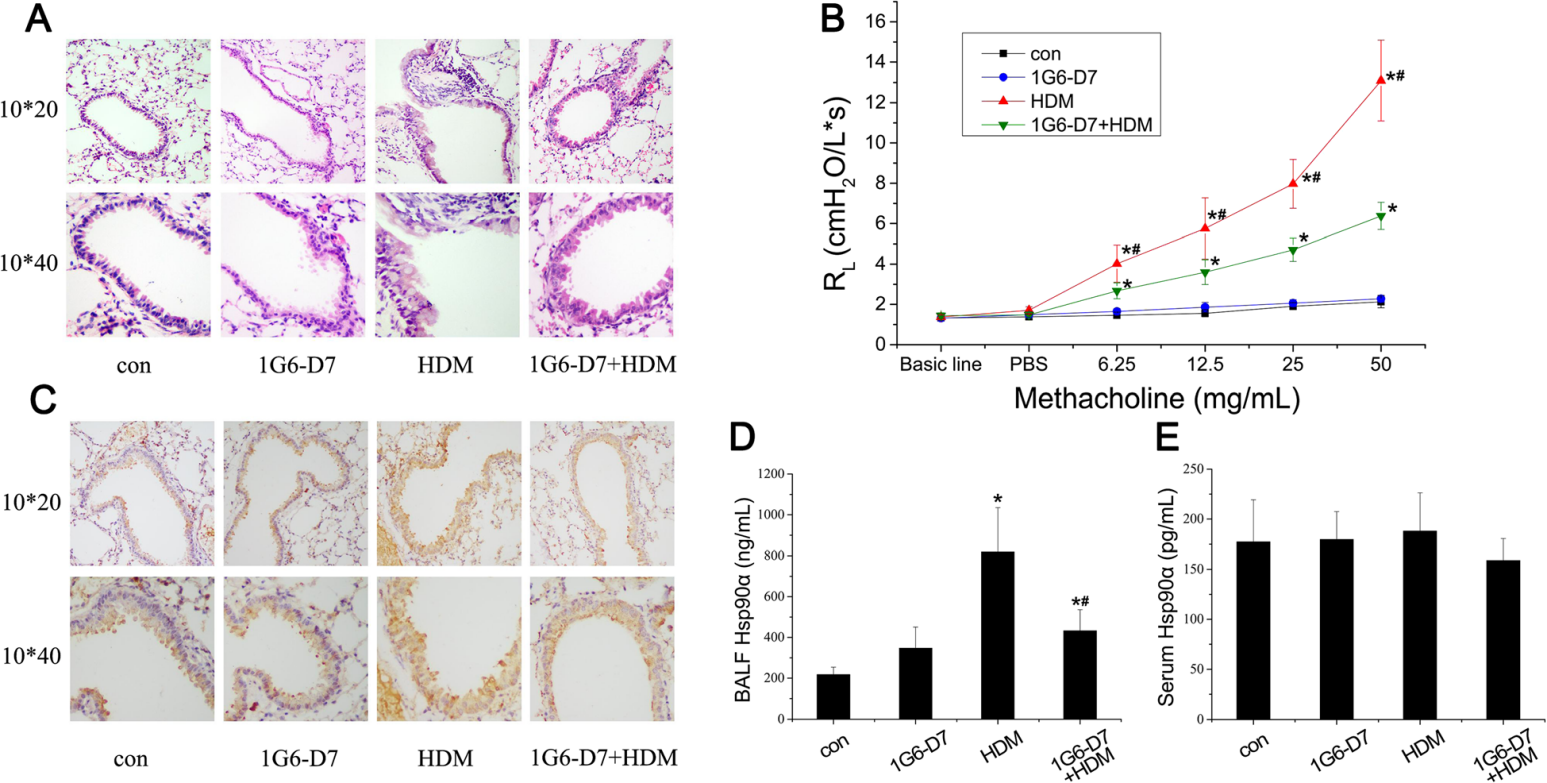
HDM induced the expression of secreted Hsp90α in asthmatic mice and the effect of 1G6-D7 on animal models. **a**: Lung sections were stained with H&E.Magnification,200 (top panel) and 400 (bottom panel).**b**: Airway resistance (RL) was assessed 24 h after the last administration. Exponentially increasing concentration of methacholine caused higher RL in the HDM group than in con group, mice pretreated with 1G6-D7 showed a partly reduction of RL. Data shown are means±SD, (*P < 0.05 vs the control group; ^#^*P* < 0.05 vs the HDM group). **c**: Immunohistochemical staining of HSP90α (brown) in serial lung sections of asthmatic mice. Magnification,200 (top panel) and 400 (bottom panel). **d**&**e**: The secretion of Hsp90α in BALF and serum samples from mice (*n* = 8) was assessed by ELISA. Data shown are means±SD, (*P < 0.05 vs the control group;* ^#^*P* < 0.05 vs the HDM group)

Histopathology revealed markedly large numbers of infiltrating inflammatory cells in the peribronchial regions, as well as visible epithelial hyperplasia and a degree of epithelial shedding while 1G6-D7, a highly selective Hsp90α inhibitor with a concentration of 25μg/ml, partially prevented the HDM-induced response (Fig. 1a). Lung function showed an increased airway resistance values in HDM group which was dose-dependently provocated by methacholine. The effects of HDM were blocked by co-administration of 1G6-D7 (Fig. 1b). Immunohistochemistry revealed the present of Hsp90α in the epithelial cells, with an increased signal in the epithelial cells in the HDM-induced lungs of asthmatic mice, and this increase was prevented by 1G6-D7 (Fig. 1c). Furthermore, we analysed the BALF and serum collected on the day 56 and observed a significant upregulation of Hsp90α, and the co-administration of 1G6-D7 could suppress this response in the BALF (Fig. 1d&e). Taken together,these results suggested that HDM promoted AHR, injury of epithelium, and airway inflammation via a secreted Hsp90α-increase dependent mechanism.

### Secreted Hsp90α participated in epithelial barrier dysfunction of asthmatic mice

**Fig. 2:**
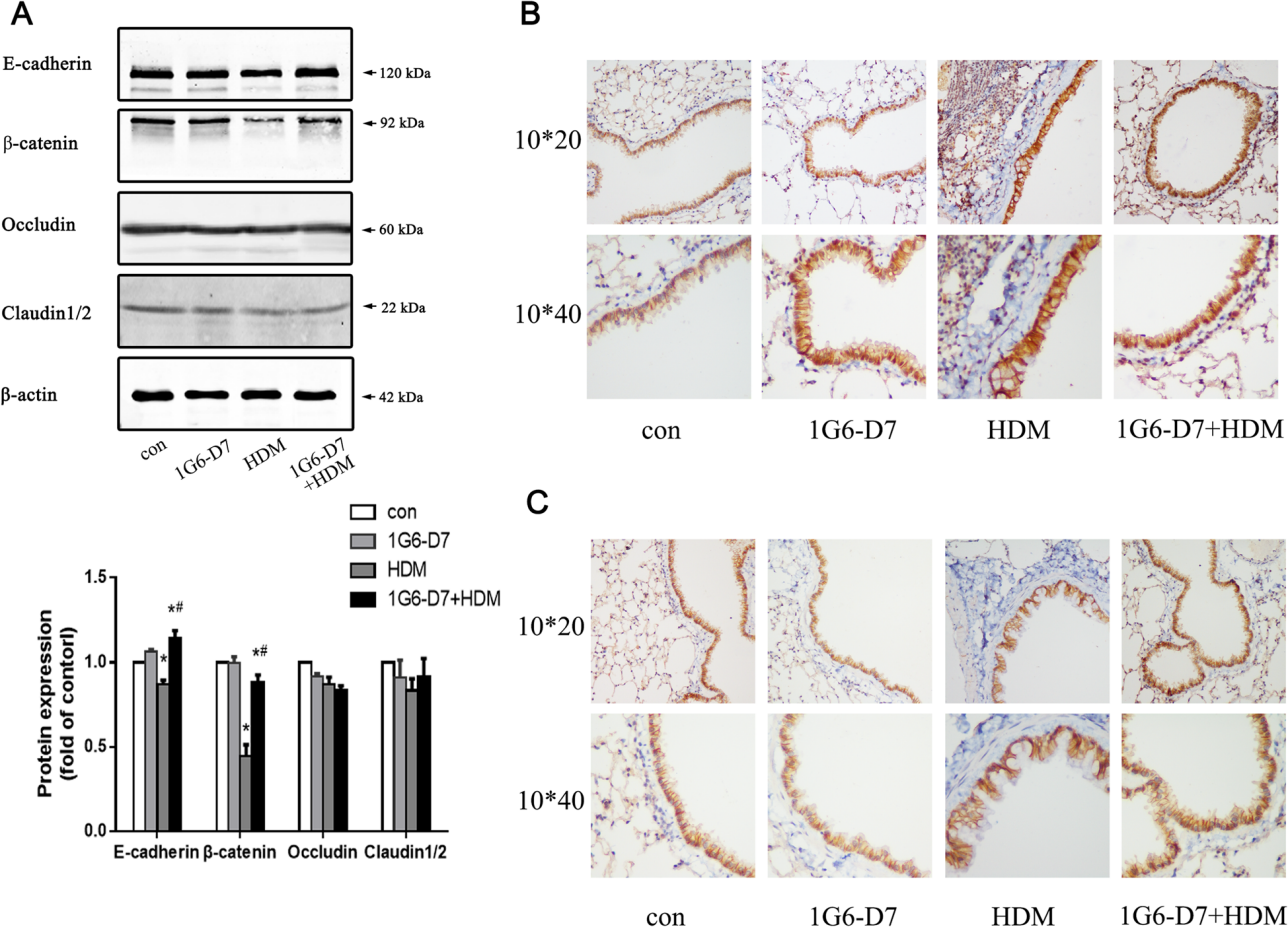
The effect of 1G6-D7 on barrier proteins disruption and expressions in asthmatic mice. **a**: Western blotting analysis of barrier proteins. Data shown are means±SD, (*P < 0.05 vs the control group;* ^#^*P* < 0.05 vs the HDM group). **b**: Immunohistochemical staining of E-cadherin (brown) in serial lung sections of asthmatic mice. Magnification,200 (top panel) and 400 (bottom panel). **c**:Immunohistochemical staining of β-catenin (brown) in serial lung sections of asthmatic mice. Magnification,200 (top panel) and 400 (bottom panel)

The integrity of epithelial barrier proteins is an essential first line of defense that deters permeability of a variety of factors from the outside environment. Therefore, changes in expression of these proteins, including E-cadherin and β-catenin, are also likely to be a central feature of the restoration of epithelial function by 1G6-D7 in the murine asthma model. Western bolts showed a downregulation of E-cadherin and β-catenin in the asthmatic lungs by HDM stimulation, this response were blocked by co-administration of 1G6-D7; However, asthmatic mice showed no changes of both Occludin and Claudin 1–2 when stimulated by HDM and treated with 1G6-D7.(Fig. 2a). Immunohistochemistry exhibited a conspicuous disruption and dislocation of E-cadherin and β-catenin in epithelial cells in the lungs of HDM-induced mice compare with sham controls, which were ameliorated by co-administration of 1G6-D7.(Fig. 2b &c). Considering the above, these results suggested that secreted Hsp90α affected the expression and integrity of AJs in asthmatic mice.

### Secreted Hsp90α promoted the release of Th2 cytokines in asthmatic mice

**Fig. 3:**
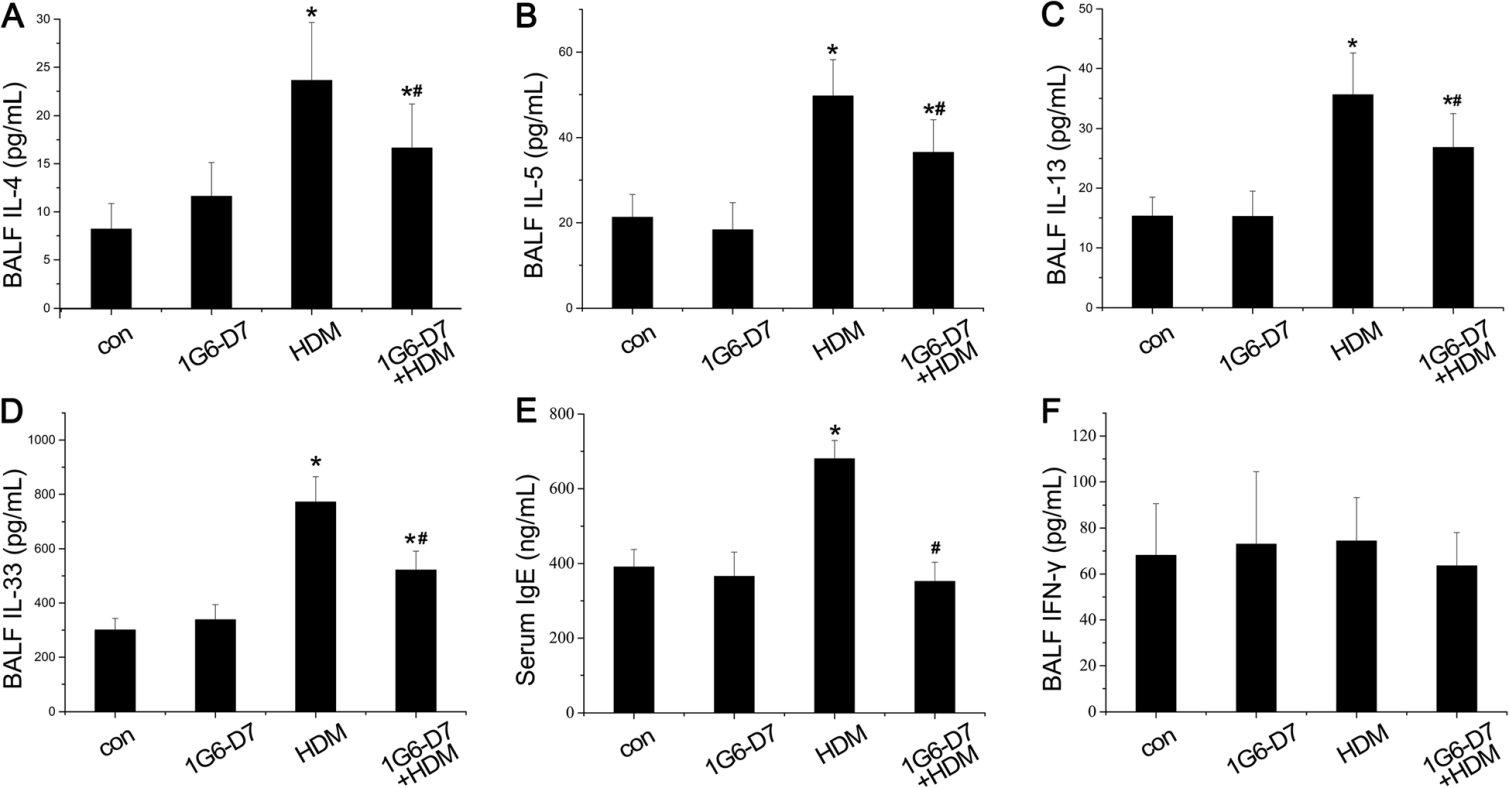
The effect of 1G6-D7 on HDM-induced release of cytokines and serum IgE. **a**-**d**:IL-4(n = 8), IL-5(n = 8), IL-13(n = 8), and IL-33(n = 8) in BALF were assessed by ELISA. **e**: IgE(n = 8) in serum was assessed by ELISA. **f**: IFN- γ (n = 8) in BALF was assessed by ELISA. Data shown are means±SD, (*P < 0.05 vs the control group;* ^#^*P* < 0.05 vs the HDM group)

Imbalance in Th1/Th2 immunoregulation is an vital characteristic in asthma, along with airway inflammation. Next, we directed our study towards the effect of 1G6-D7 on HDM-induced airway inflammation of asthmatic mice. HDM stimulation led to increased levels of IL-4, IL-5 and IL-13 in BALF of asthmatic mice. This response were blocked by co-administration of 1G6-D7(Fig. 3a,b&c). ELISA showed a significant upregulation of IL-33 in BALF which was regarded as a cytokine to stimulate the release of Th2 cytokines in mast cells, lymphocytes and eosinophils. Additionally, a pronounced overexpression of IgE in serum was observed in asthmatic mice compared with sham controls. Both increase were reduced to control levels by treatment of 1G6-D7 (Fig. 3d&e). The expression of IL-4, IL-5, IL-13 and IL-33 in BALF were not sensitive to 1G6-D7 alone (Fig. 3a,b,c&d). However, IFN-γ expression in BALF was neither sensitive to HDM stimulation nor 1G6-D7 treatment (Fig. 3f). Giving the above findings, it indicated that HDM-induced airway inflammation was a local and Th2 advantaged inflammation and 1G6-D7 might be a therapeutic strategy to ameliorate Th2 inflammation in vivo.

### HDM promoted secretion of Hsp90α both in 16HBE cells and Sh-Hsp90α 16HBE cells

**Fig. 4:**
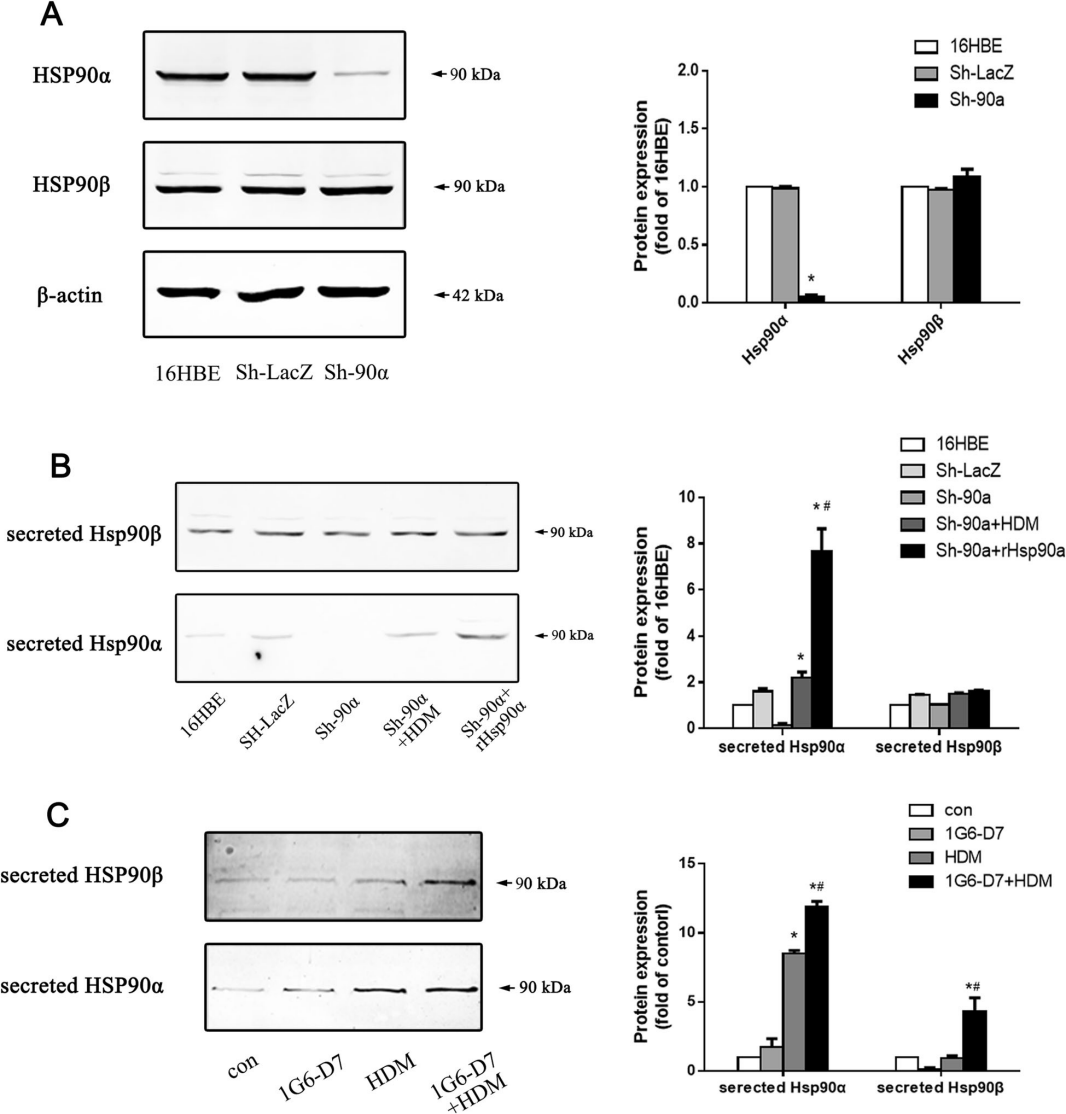
The secretion of Hsp90α in Hsp90α-knockdown cells and normal 16HBE cells. **a**: The expression of Hsp90α in Hsp90α-knockdown cells was assessed by western blots. Data shown are means±SD, (*P < 0.05 vs the control group,*n* = 3). **b**: Western blotting analysis of secreted Hsp90α and Hsp90β in Hsp90α-knockdown cells. Data shown are means±SD, (*P < 0.05 vs the control group,* ^#^*P* < 0.05 vs the HDM group,n = 3). **c**: Western blotting analysis of secreted Hsp90α and Hsp90β in normal 16HBE cells. Data shown are means±SD, (*P < 0.05 vs the control group,* ^#^*P* < 0.05 vs the HDM group,n = 3)

To study the role of secreted Hsp90α in asthmatic airway epithelial barrier, we employed RNAi in 16HBE cell line (airway epithelial cell) to interfere the expression of Hsp90α. Western blots showed an almost absence of Hsp90α (but no Hsp90β) in the isolated cell clone following infection, which demonstrated the Hsp90α-knockdown cell model was successfully constructed (Fig. 4a&b). HDM stimulation led to increased expression of secreted Hsp90α in Hsp90α-knockdown cells and normal cells,whereas the expression levels of secreted Hsp90α in normal cells was intenser (Fig. 4b&c). The treatment of hrHsp90α led to a significant upregulation of secreted Hsp90α in Hsp90α-knockdown cells (Fig. 4b). Giving these facts, the results showed that HDM stimulation promoted the expression of secreted Hsp90α in airway epithelial cell.

### 1G6-D7 attenuated HDM-induced bronchial epithelial hyperpermeability

**Fig. 5:**
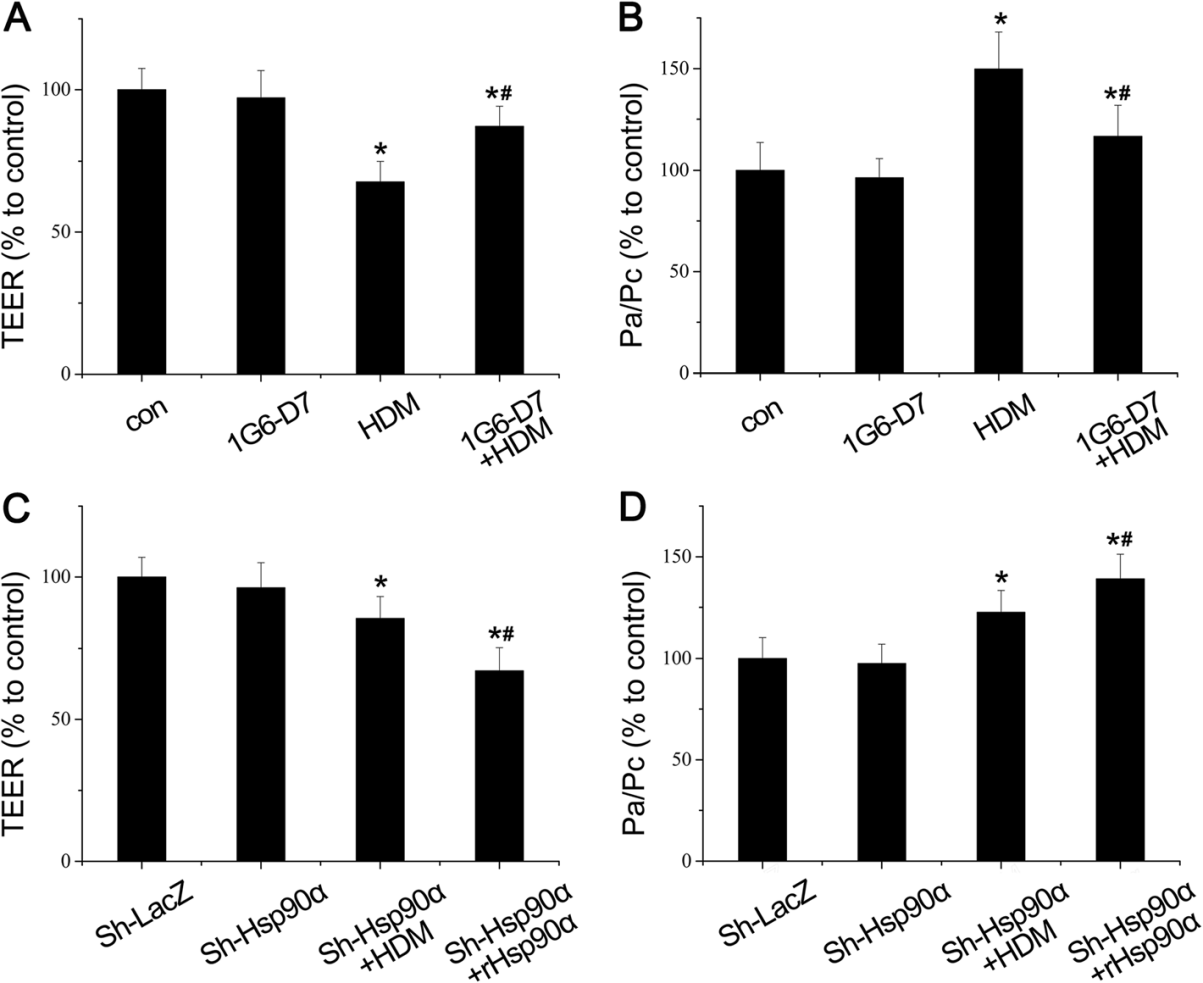
The effect of 1G6-D7 and hrHsp90α on bronchial epithelial hyperpermeability in 16HBE cells. **a**: The transepithelial electrical resistance (TEER) was measured in normal 16HBE cells. Data shown are means±SD, (*P < 0.05 vs the control group,* #*P* < 0.05 vs the HDM group,n = 3). **b**: The Permeability (FITC-dextran) was measured in normal 16HBE cells. Data shown are means± SD, (*P < 0.05 vs the control group,* ^#^*P* < 0.05 vs the HDM group,n = 3). **c**: The transepithelial electrical resistance (TEER) was measured in Hsp90α-knockdown cells. Data shown are means± SD, (*P < 0.05 vs the control group,* ^#^*P* < 0.05 vs the HDM group,n = 3). **d**: The Permeability (FITC-dextran) was measured in Hsp90α-knockdown cells. Data shown are means±SD, (*P < 0.05 vs the control group,* ^#^*P* < 0.05 vs the HDM group,n = 3)

Having observed that the restoration of epithelial function by 1G6-D7 in vivo, we aimed to find out whether secreted Hsp90α resulted in hyperpermeability of bronchial epithelial cells in vitro. The epithelial barrier function showed a decrease of TEER in normal cells and Hsp90α-knockdown cells by HDM stimulation ditectly, whereas the decrease levels of TEER in Hsp90α-knockdown cells was slight. The co-administration of 1G6-D7 could suppress this response in normal cells,while the co-administration of hrHsp90α led to a stronger decrease in Hsp90α-knockdown cells (Fig. 5a&c). Besides this, the FITC-dextran permeability were increased in normal cells and Hsp90α-knockdown cells influenced by HDM stimulation (Fig. 5b&d). The increased levels of FITC-dextran permeability in normal cells was stronger, while it was prevented by treatment of 1G6-D7.And the co-administration of hrHsp90α could enhance the decrease level of FITC-dextran permeability in Hsp90α-knockdown cells (Fig. 5b&d). Considering all these, these results implied that secreted Hsp90α directly participated in the epithelial cells dysfunction and more extracellular Hsp90α lead to a worse effect.

### 1G6-D7 partly restored the HDM-induced disorder of E-cadherin and β-catenin

**Fig. 6:**
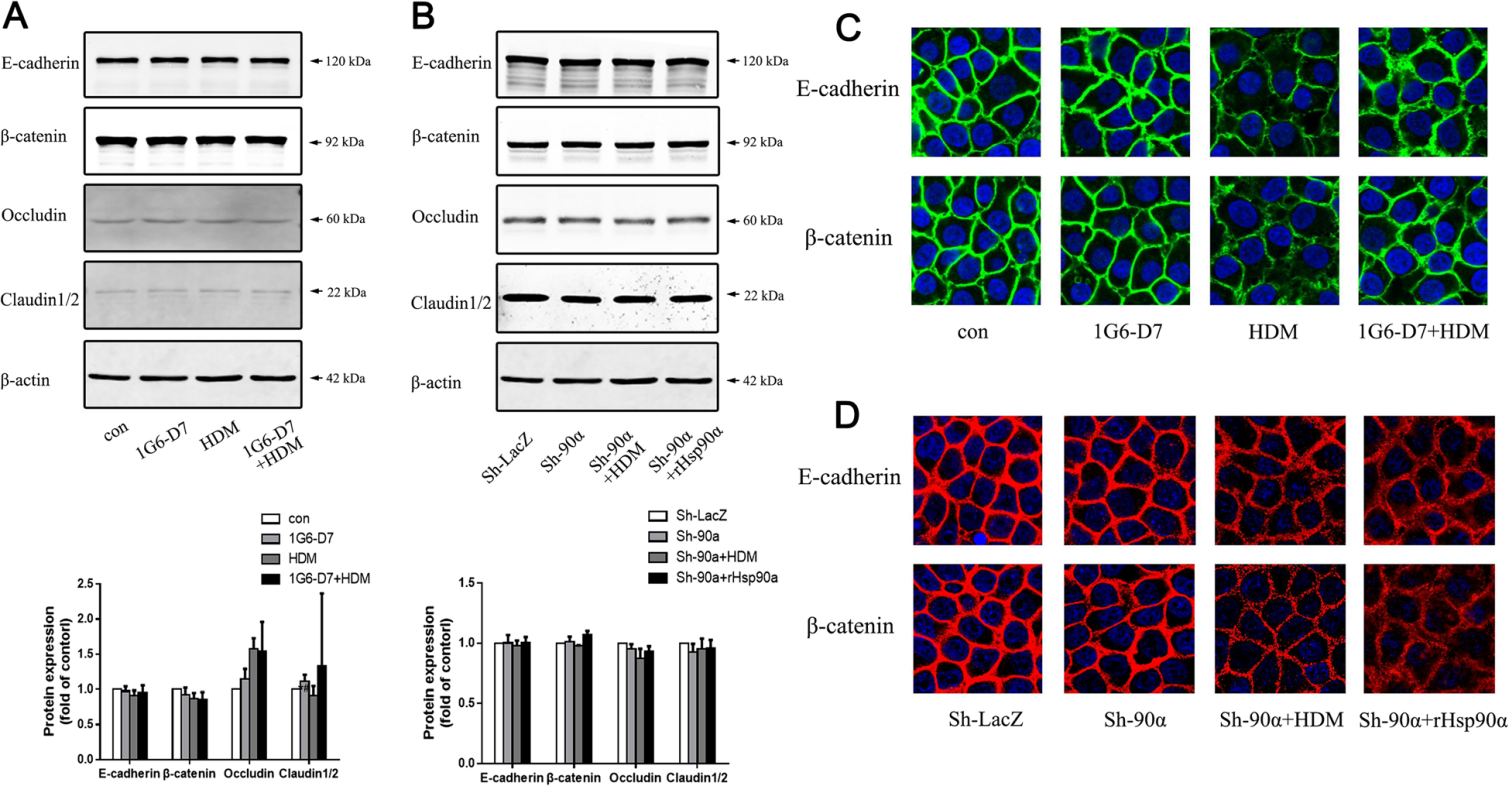
The effect of 1G6-D7 on epithelial barrier proteins in HDM-induced normal 16HBE cells and Hsp90α-knockdown 16HBE cells. **a**: Western blotting analysis of barrier proteins in normal 16HBE cells. Data shown are means± SD,*n* = 3. **b**: Western blotting analysis of barrier proteins in Hsp90α-knockdown cells. Data shown are means±SD,n = 3. **c**:Immunofluorescence of E-cadherin (top panel) and β-catenin (bottom panel) in normal 16HBE cells. **d**:Immunofluorescence of E-cadherin (top panel) and β-catenin (bottom panel) in Hsp90α-knockdown cells

Having demonstrated that secreted Hsp90α led to hyperpermeability of bronchial epithelial cells in vitro, we questioned whether it involved the disorder of E-cadherin and β-catenin in epithelial barrier. Western blotting analysis revealed that HDM treatment did not affect the expression of E-cadherin or β-catenin in normal 16HBE cells and Hsp90α-knockdown cells (Fig. 6a&b). Expression of E-cadherin or β-catenin were not sensitive to 1G6-D7 treatment nor hrHsp90α stimulation (Fig. 6a&b). However, the immunofluorescence showed that HDM promoted delocalization of E-cadherin and β-catenin both in normal 16HBE cells and Hsp90α-knockdown cells, exhibiting discontinuous and diffusing from the adjacent cell borders to cytoplasm (Fig. 6c&d). The delocalization of epithelial barrier was partly abrogated by 1G6-D7 in vitro (Fig. 6c), while hrHsp90α led to a more signficant disorder of E-cadherin and β-catenin in Hsp90α-knockdown cells (Fig. 6d). These results proved that secreted Hsp90α not only affected the barrier function in cells but also caused the dislocation of AJs directly.

### 1G6-D7 reduced the expression of p-AKT, p-ERK1/2, p-P38 and LRP-1 induced by HDM stimulation

**Fig. 7:**
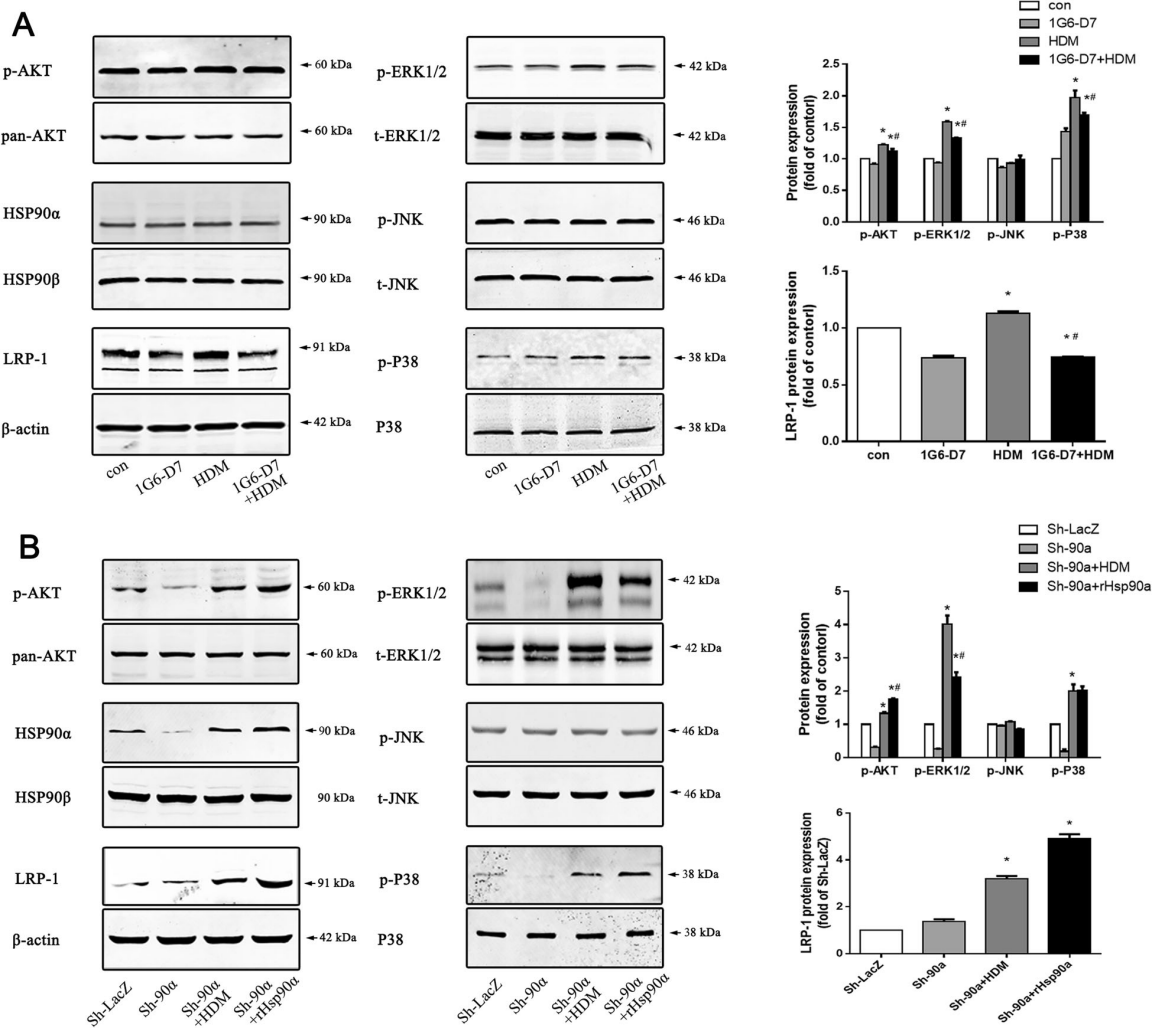
The effect of secreted Hsp90α on asthmatic downstream pathway factors and the impact of 1G6-D7 on HDM-induced activation of AKT, ERK, and P38. **a**:The activation of LRP-1, p-AKT (Thr 308), p-ERK1/2 and p-P38 in normal 16HBE cells were evaluated by western blots. Data shown are means±SD, (*P < 0.05 vs the control group,* ^#^*P* < 0.05 vs the HDM group,n = 3). **b**: The activation of LRP-1, p-AKT (Thr 308), p-ERK1/2 and p-P38 in Hsp90α-knockdown cells were evaluated by western blots. Data shown are means±SD, (*P < 0.05 vs the control group,* ^#^*P* < 0.05 vs the HDM group,n = 3)

It is known that activation of the AKT, ERK, and P38 pathways participate in dysfunction of airway epithelial barrier. Based on the fact that secreted Hsp90α inhibition ameliorated asthmatic epithelial function and epithelial barrier, we investigated the relationship between signaling proteins and secreted Hsp90α. Western blots exhibited that HDM promoted the phosphorylation of AKT (Thr 308), ERK1/2 and P38 both in normal 16HBE cells and Hsp90α-knockdown cells, while there were no changes in JNK (Fig. 7a&b). In addition, under the HDM stimulation, it showed a significant upregulation of LRP-1 both in normal 16HBE cells and Hsp90α-knockdown cells,which was acknowledged as the receptor of extracellular Hsp90α(Fig. 7a&b). Furthermore, administration of 1G6-D7 suppressed these response in normal 16HBE cells (Fig. 7a). Treatment with hrHsp90α caused notable the phosphorylation of AKT (Thr 308),while compared with stimulation of HDM in Hsp90α-knockdown cells that achieved the phosphorylation of AKT (Thr 308), ERK1/2 and P38,(Fig. 7b). Taken together,these suggested that secreted Hsp90α promoted the disorder of airway epithelial barrier by inducing phosphorylation of AKT, ERK and P38 via LRP-1.

### Secreted Hsp90α promoted barrier dysfunction via PI3K/AKT pathway

**Fig. 8:**
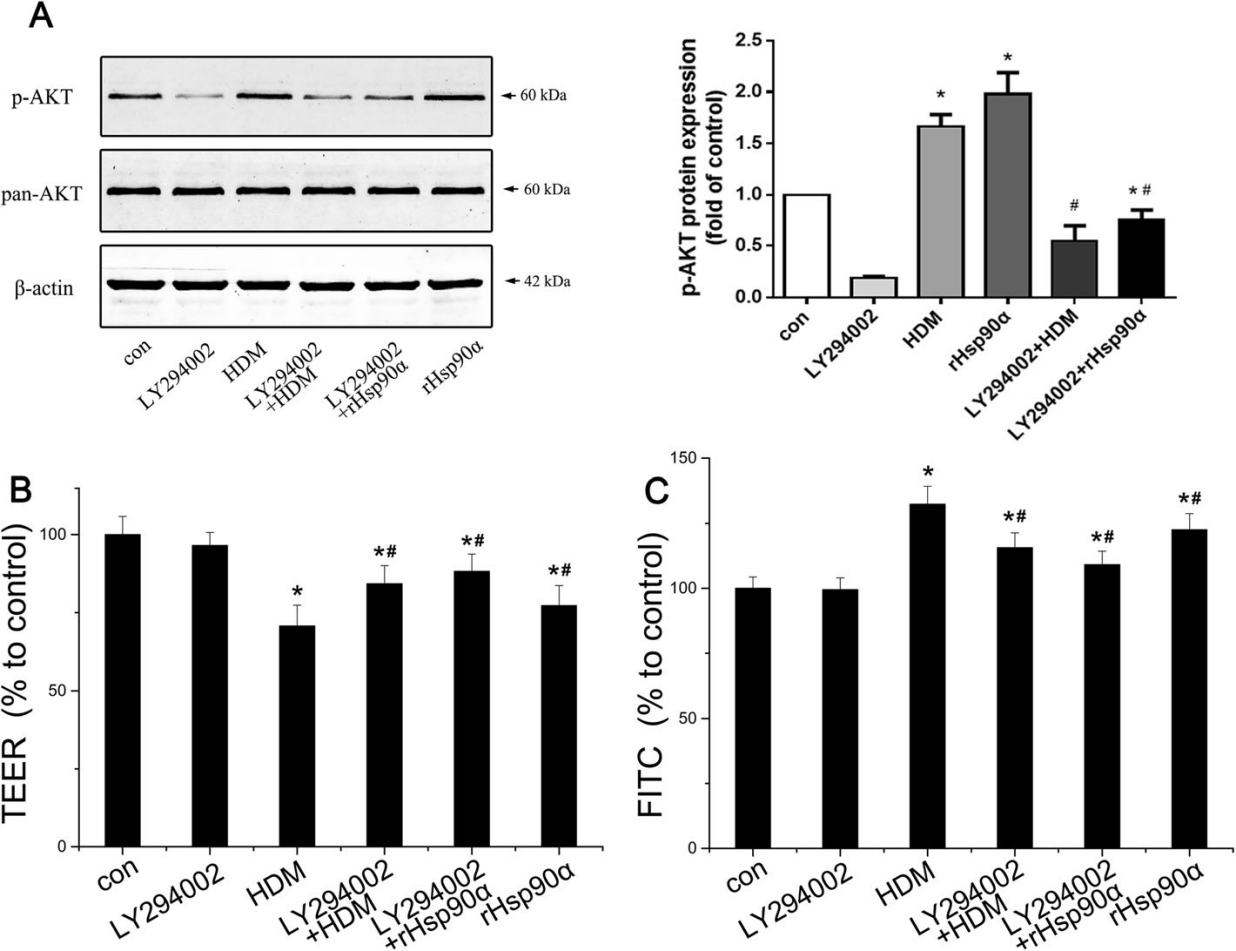
The effect of LY294002 on the activation of AKT induced by HDM and hrHsp90α in normal 16HBE cells. **a**:Western blotting analysis of the activation of AKT in normal 16HBE cells.(*P < 0.05 vs the control group,* ^#^*P* < 0.05 vs the HDM group/the hrHsp90α group,n = 3). **b**:The transepithelial electrical resistance (TEER) was measured in normal 16HBE cells. Data shown are means±SD, (*P < 0.05 vs the control group,* ^#^*P* < 0.05 vs the HDM group/the hrHsp90α group,n = 3). **c**:The transepithelial electrical resistance (TEER) was measured in normal 16HBE cells. Data shown are means±SD, (*P < 0.05 vs the control group,* ^#^*P* < 0.05 vs the HDM group,n = 3)

As we demonstrated above, secreted Hsp90α caused phosphorylation of AKT via LRP-1 to mediate the dysfunction of airway epithelial barrier. In our previous study, PI3K/AKT pathway had been proved to be important in epithelial dysfunction induced by HDM. Therefore, we focused on whether secreted Hsp90α leads to barrier dysfunction by activing PI3K/AKT pathway. The phosphorylation of AKT was inhibited by LY294002, a highly selective PI3K inhibitor with a median inhibitory concentration of 1.4uM in a cell-free assay. We determined to use 10 μM LY294002 based on pilot studies. Western blots exhibited that both stimulation of HDM and hrHsp90α led to the phosphorylation of AKT (Thr 308), which could be suppressed by LY294002(Fig. 8a). The epithelial barrier function showed a significant elevation of TEER and a remarkable reduction of FITC-dextran permeability in normal 16HBE cells by co-administration of LY294002, indicating the restoration of epithelial function (Fig. 8b&c). These results implied secreted Hsp90α promoting the HDM-induced barrier dysfunction partly by activating PI3K/AKT pathway.

## Discussion

In this study, we proved that secreted Hsp90α participated in HDM-induced dysfunction of epithelial barrier, airway resistance and airway inflammation, the function was partly mediated by the PI3K/AKT pathway. 1G6-D7 protected AECs from dysfunction and downstream signaling transduction. The study aimed to offer a potential therapeutic strategy to asthma.

Secreted Hsp90α participated in many diseases like tumor, inflammation [8, 10, 17, 18]. Previous research had demonstrated that Hsp90α and Hsp90β mRNA expressions were increased in peripheral blood mononuclear cells of patients with asthma [19]. Our research confirmed the association between asthma and secreted Hsp90α [20]. Based on the results of our study, we did further research in the mechanisms of secreted Hsp90α in asthma.

Asthma is characterized by airway epithelial barrier dysfunction, Th2-mediated airway inflammation, airway remodeling and AHR [5, 21, 22]. The integrity of the airway epithelial barrier is dependent on cellular integrity and strong cell-cell adhesion mediated by particular junctions [23, 24]. Specifically, E-cadherin complexes with β-catenin to form an adhesive junction (AJ) that is involved in signal transduction, providing the structural support required to form these ligation complexes [6, 14, 22, 25–30]. We found secreted Hsp90α was induced by HDM and participated in the disruptive effect of HDM in mice and AECs. Furthermore, 1G6-D7,a monoclonal antibody developed in Wei Li’s laboratory and specifically combined to secreted Hsp90α [15, 25–27],showed protective effects on barrier function and proteins in vitro and in vivo. The intracellular signaling pathways, functions of molecular chaperone and intracellular micro-environment were not affected by 1G6-D7 directly since it’s molecular was too big to enter the cells. We found that HDM-induced dislocations of E-cadherin and β-catenin manifesting by ICF and IHC, while 1G6-D7 had protective effects on these changes. Furthermore, after knocking down Hsp90α, exogenous supplement of human recombinant Hsp90α also caused severe dislocation of E-cadherin and β-catenin. All the above indicated that secreted Hsp90α mignt function in promoting cell migration and inflammation, therefore accelerating the loss of the cell-cell junctions. However, it is worth thinking that HDM reduced the expressions of E-cadherin and β-catenin in mice but not in 16HBE cells, suggesting that there might be more mechanisms to be found in vivo.

In our previous research, we found that Hsp90α is involved in HDM-induced asthmatic airway epithelial barrier disruption [20]. Secreted Hsp90α can be induced by stress proteins and cytokines, which play a key role in inflammation and barrier function [31–37].. We found that the expression of BALF Hsp90α had been promoted by HDM, while 1G6-D7 reduced this response. Furthermore, we found chronic HDM-stimulation provoked a robust Th2 response accompanied by more modest changes in IFN-γ levels in mice, suggesting a shift in the Th1/Th2 balance toward Th2, which was an vital characteristic in asthma. The blockade of secreted Hsp90α with 1G6-D7 reduced the release of HDM-induced Th2 cytokines. IL-4 was important in CD4+ lymphocyte differentiation and the production of IgE,while IL-13 drived airway hyperresponsiveness, mucus production, and subepithelial fibrosis. IL-5 was an obligate cytokine for the survival and maturation of eosinophils. IgE has a positive correlation with eosinophil inflammation while IL-33 could strongly promote the release of Th2 cytokines [38, 39].Co-administration of 1G6-D7 dramatically reduced serum IgE and BALF IL-33 levels as well as HDM-induced Th2 inflammation stimulated by decreasing the levels of IL-4,IL-5,IL-13. The mechanism of secreted Hsp90α in immune-regulation has not been studied in this report,though in previous study, Hsp90 has been proved to be vital in innate immunity and the antigens cross-presentation [40]. In our study, 1G6-D7 only combined to Hsp90α,ameliorated the Th2 inflammation and airway resistance of asthmatic mice. The impact of extracellular Hsp90α in immune-regulation had already been confirmed in activation of monocytes and other pathological processes [41]. More should be done to prove the role of secreted Hsp90α in asthmatic inflammation and immune-regulation.

Our previous studies had revealed that the signaling pathway of VEGF, PI3K/AKT, MAPK/ERK mediated the dysfunction of AECs [11–13, 42]. The relationship between AKT, ERK and secreted Hsp90α has not been clearly demonstrated. Studies indicated that cells under a variety of stimulations could actively secrete Hsp90α.It has been confirmed that secreted Hsp90α binded to LRP-1, subsequently activated downstream signaling pathway and eventually caused a series of signal molecules change [10, 18, 43]. In this report, the effect of HDM in promoting activation of LRP-1 and phosphorylation of AKT, ERK and P38 were in accordance with our previous studies, while the co-administration of 1G6-D7 reduced the activation of these proteins. But a supplement of hrHsp90α showed severe effect on AKT signaling molecules in Hsp90α-knockdown cells. Although JNK/p-JNK was reported to associate with asthma, but there were no changes in our data. JNK was a client protein of Hsp90α and the 1G6-D7 had no effect on it, even hrHsp90α did not increase the phosphorylation of JNK. Phosphorylation of JNK usually mediates via TGF-β pathway, but it does not involve in secreted Hsp90α [44]. Recently, it has been proved that AKT was crucial to secreted Hsp90α as it works in various cellular process such as promoting cell motility and wound healing [45]. Therefore, we kept on focusing on the relationship between secreted Hsp90α and PI3K/AKT pathway, and the latter had been proved to be important in epithelial dysfunction induced by HDM in our previous research [13, 42]. As we could see, a PI3K inhibitor LY294002 used in this study protected the AECs from hrHsp90α and HDM induced decrease of TEER and increase of FITC dextran permeability, suggesting that secreted Hsp90α played an important role in dysfuction of airway epitheliel barrier via promoting the phosphorylation of AKT, and the PI3K/AKT pathway was exactly the downstream of it. These results were in accordance with Wei Li′ article, which confirmed the signaling pathway of secreted Hsp90α - LRP-1 - p-AKT in HDFs [45]. But it was strange that Wei Li proved the phosphorylation of AKT was Ser473 but not Thr308, whereas in this report and in our previous studies, we found phosphorylation of AKT at Thr308 played an important role in epithelial dysfunction. Moreover, secreted Hsp90α promoted the activation of AKT at Thr308 but the treatment of 1G6-D7 prevented it in vitro. All the above indicated that secreted Hsp90α promoted the development of asthma by inducing epithelial barrier dysfunction via PI3K/AKT pathway.

## Conclusion

In summary, we demonstrated that HDM-induced asthmatic mice and 16HBE cells increased the secretion of Hsp90α, which played an important role in asthma since it induced the epithelial barrier dysfunction via PI3K/AKT pathway. Neutralization of Secreted Hsp90α by 1G6-D7 inhibited the phosphorylation of AKT and ameliorated the bronchial epithelial barrier dysfunction induced by HDM. Therefore, the anti-secreted Hsp90α therapy might be a potential treatment in future.

## Data Availability

The data set during and/or analyzed during the current study available from the corresponding author on reasonable request.

## Abbreviations

16HBE: Human bronchial epithelial cell line 16HBE14o-
AECs: Airway epithelial cells
AHR: Airway hyperresposiveness
AJs: Adherens junctions
DAPI: 4′,6-diamidino-2-phenylindole dihydrochloride
FITC-dextran: FITC-dextran flux
HDM: House dust mite
Hr: Human recombinant
HSP: Heat shock protein
IHC: Immunohistochemistry
One-way ANOVA: One-way analysis of variance
RL: Lung resistance
TDI: Toluene diisocyanate
TEER: Transepithelial electrical resistance
TJs: Tight junctions
